# Successful treatment of relapsed chronic lymphocytic leukemia with venetoclax in a patient with severe chronic kidney disease

**DOI:** 10.1002/ccr3.5735

**Published:** 2022-04-14

**Authors:** Hiroyuki Sugiura, Nobuo Sezaki, Tatsunori Ishikawa, Taiga Kuroi, Sachiyo Okamoto, Naho Nomura, Taro Masunari, Yukio Nakasako, Toru Kiguchi, Mitsune Tanimoto

**Affiliations:** ^1^ Department of Hematology Chugoku Central Hospital of Japan Mutual Aid Association of Public School Teachers Fukuyama Japan; ^2^ Department of Diabetology and Nephrology Chugoku Central Hospital of Japan Mutual Aid Association of Public School Teachers Fukuyama Japan; ^3^ 26263 Department of Diabetes Endocrinology and Hematology Dokkyo Medical University Saitama Medical Center Koshigaya Japan

**Keywords:** chronic lymphocytic leukemia, severe chronic kidney disease, tumor lysis syndrome, venetoclax

## Abstract

Venetoclax is a promising new drug for relapsed or refractory chronic lymphocytic leukemia (CLL). However, venetoclax use had not been reported in severe chronic kidney disease (CKD) patients. We report the first case of relapsed CLL in a severe CKD patient that was successfully treated with venetoclax.

## Graphical abstract

MR perfusion can be used to distinguish intracranial Rosai‐Dorfman disease from meningioma, and permit effective nonoperative treatment.

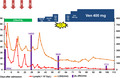

## 1 INTRODUCTION

Chronic lymphocytic leukemia (CLL) is a chronic lymphoproliferative disorder characterized by progressive accumulation of functionally incompetent lymphocytes that are usually monoclonal in origin.^1^ Most patients will respond to initial therapy, but disease relapse invariably occurs and becomes refractory with each relapse. Venetoclax, which is an orally bioavailable, selective, small‐molecule inhibitor of BCL2, is a new therapeutic agent for relapsed or refractory CLL.^2^ Concerning the toxicity of venetoclax, tumor lysis syndrome (TLS) has been reported as a severe adverse event due to venetoclax treatment and intensive prevention and strict monitoring are recommended according to the risk level of TLS defined by tumor burden and renal function for the use of venetoclax.^3^

Chronic kidney disease (CKD) refers to kidney damage or decreased kidney function for three or more months, regardless of the cause. Decreased kidney function is defined as a glomerular filtration rate (GFR) of <60 ml/min/1.73 m^2^, and severe CKD (stage 4) is defined as a GFR of <30 ml/min/1.73 m^2^.^4^ To the best of our knowledge, venetoclax use has not been reported in patients with severe CKD. Herein, we describe a case of relapsed CLL patient with severe CKD due to congenital solitary kidney that was successfully treated with venetoclax.

## 2 CASE DESCRIPTION

A 78‐year‐old man with CKD and congenital solitary kidney was diagnosed with CLL 15 years prior. The Rai classification was intermediate 1, the Binet classification was A, and watchful waiting was adapted. However, lymphocytosis and lymph node enlargement progressed 4 years after his first visit; therefore, he was prescribed oral fludarabine treatment. Four years after the oral fludarabine treatment, lymph node enlargement progressed again and hence he was prescribed bendamustine with rituximab (BR) therapy. Treatment response was good, but because severe neutropenia occurred, BR therapy was stopped at two cycles. Two years after the BR therapy, CLL progressed again, and BR treatment was administered again for two cycles. Two years after the last BR treatment, lymphocytosis progressed again; therefore, ibrutinib, an inhibitor of Bruton's tyrosine kinase and an effective agent for CLL, was initiated and administered for 2 years. However, lymphocytosis and lymph node enlargement gradually progressed, and he was admitted to our hospital.

**FIGURE 1:**
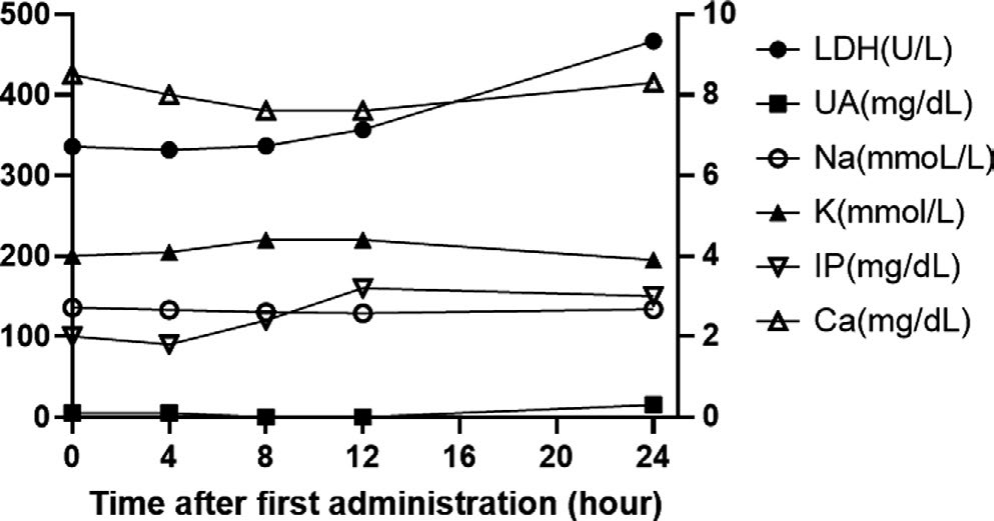
Transition of laboratory data on the first day of administration of venetoclax. LDH and Na follow the left axis, and other markers follow the right axis. Ca, calcium; IP, inorganic phosphorus; K, potassium; LDH, lactate dehydrogenase; Na, sodium; UA, uric acid

**FIGURE 2:**
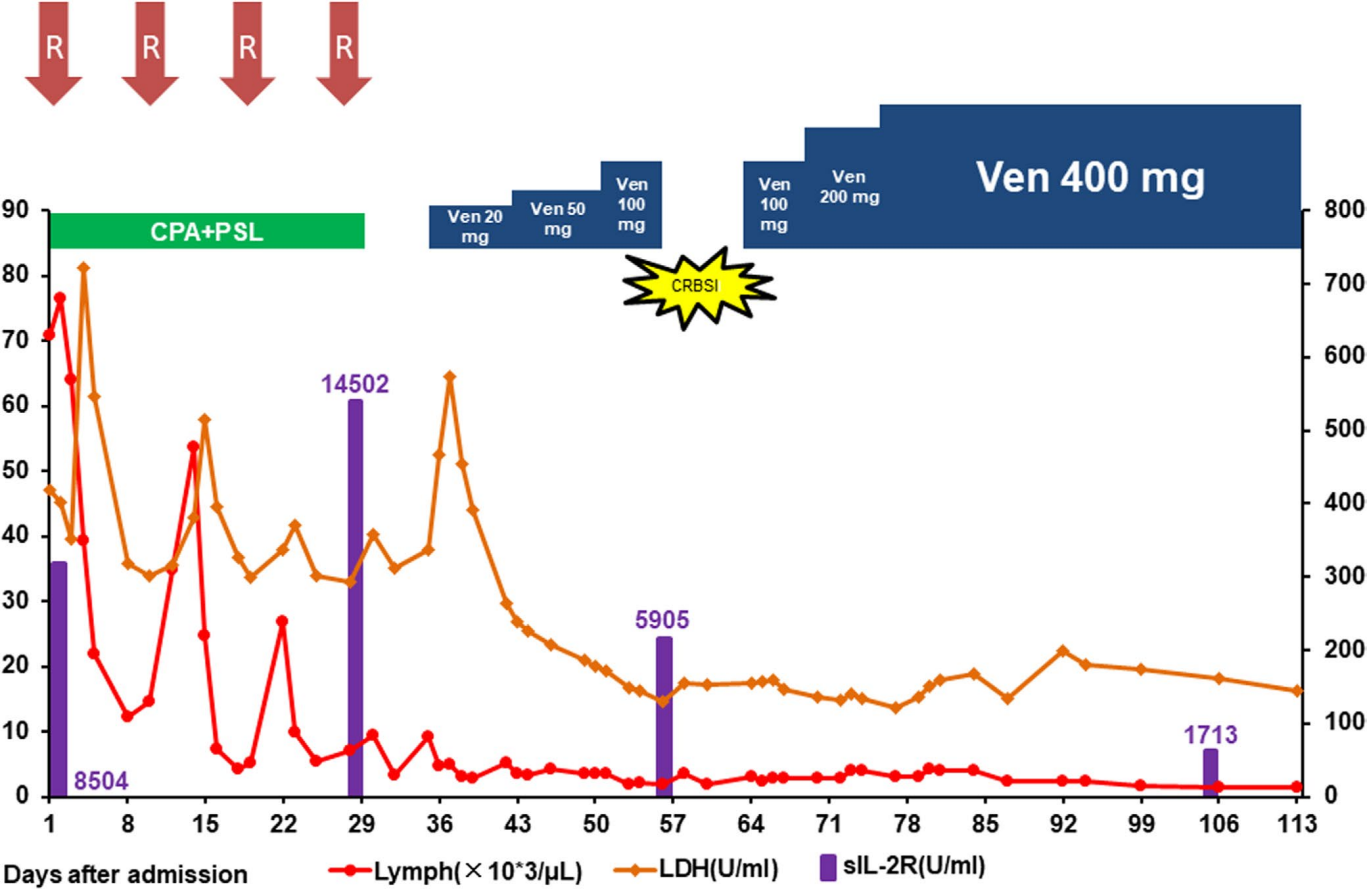
Clinical course of the patient from the admission day to the discharge day (Day 113). Lymphocyte follows left axis and LDH follows right axis. After debulking by CPA + PSL + Rituximab, venetoclax was started, and the dose was increased gradually; the treatment response was good. Because of the complications of CRBSI, venetoclax treatment was stopped. After the exchange of the catheter and antibiotic therapy, venetoclax was restarted safely. CPA, cyclophosphamide; CRBSI, catheter‐related bloodstream infection; PSL, prednisolone; R, rituximab; Ven, venetoclax

Laboratory data on the admission day revealed severe lymphocytosis, severely impaired renal function, and mildly increased liver enzymes (Table 1). The serum creatinine level was 2.7 mg/dl, and the estimated GFR from creatinine was 18.7 ml/min/1.73 m^2^. He was diagnosed as having a relapse of CLL and complications of severe CKD (stage 4, GFR < 30ml/min). Computed tomography (CT) revealed systemic lymphadenopathy and splenomegaly. Because of severe lymphocytosis, systemic lymphadenopathy, splenomegaly, and severe CKD from congenital solitary kidney, the risk of TLS was considered extremely high; therefore, oral cyclophosphamide and prednisolone with four cycles of rituximab were administered to reduce tumor burden before venetoclax therapy. The lymphocyte count was reduced to 15,000 cells/μl, and lymphadenopathy and splenomegaly were ameliorated. After debulking of the tumor, 20 mg/day venetoclax was initiated, along with intensive prevention of TLS involving hydration, 60 mg/day febuxostat, and 7.5 mg/body rasburicase. This is because we assessed that the risk of TLS was still high because of the complications of severe CKD. Strict monitoring of laboratory data at 4, 8, 12, and 24 h after the start of venetoclax (Figure 1) on the first administration day revealed a slight increase in the lactate dehydrogenase level with no laboratory abnormality, which met the criteria for TLS diagnosis. Subsequently, the venetoclax dose was increased weekly to 50 mg/day and 100 mg/day without TLS, but catheter‐induced bloodstream infection occurred, and venetoclax treatment was stopped for a week. After catheter exchange and antibiotic administration, venetoclax treatment was restarted at a dose of 100 mg/day and increased weekly to 200 mg/day and 400 mg/day without TLS. Lymphocytosis and soluble interleukin‐2 receptor immediately decreased to the normal range. We observed that the response to venetoclax treatment was quite good. After venetoclax reached maintenance dose, he was discharged on Day 113 post‐admission and received additional rituximab as an outpatient (clinical course during admission is summarized in Figure 2). We told the importance of this case to patient and obtained informed consent to publish.

**TABLE 1:** Laboratory data and BMA findings on admission day

CBC and coagulation test		Biochemistry and sIL−2R	
WBC	75,360 /μl	TP	6.1 g/dl
Neu	1%	Alb	4.2 g/dl
Lymph	94%	T‐Bil	0.6 mg/dl
RBC	365 × 10^6^/μl	AST	81 U/L
Hb	10.6 g/dl	ALT	100 U/L
Plt	12 × 10^4^ /μl	LDH	418 U/L
APTT	28.5 s	γ‐GTP	144 U/L
PT‐INR	1.06	UA	7.3 mg/dl
Fib	272 mg/dl	Cre	2.7 mg/dl
D‐D	2.0 μg/ml	BUN	38 mg/dl
FDP	3.1 μg/ml	eGFRcre	18.7 ml/min/1.73
**BMA findings**		Na	139 mmol/L
NCC 52.3 × 10^4^/μl		K	5.2 mmol/L
Megakaryocyte 24/μl		Cl	103 mmol/L
Small lymphocyte 94%, CD5 +, CD20+, CD23+, light chain λ+		Ca	9.2 mg/dl
		IP	3.7 mg/dl
Chromosome analysis: 46, XY, del (11) (q?) [16], 46, XY, idem, ?t(2;3) (q21:p13) [3], 46,XY [1]		sIL‐2R	8504 U/ml

Abbreviations: Alb, albumin; ALT, alanine aminotransferase; APTT, activated partial thromboplastin time; AST, aspartate aminotransferase; BMA, bone marrow aspiration; BUN, blood urea nitrogen; Ca, calcium; CBC, complete blood count; CD, cluster of differentiation; Cl, chlorine; Cre, creatinine; D‐D, d‐dimer; eGFRcre, estimated glomerular filtration rate from creatine; FDP, fibrin degradation product; Fib, fibrinogen; Hb, hemoglobin; IP, inorganic phosphorus; K, potassium; LDH, lactate dehydrogenase; Lymph, lymphocyte; Na, sodium; NCC, nucleated cell count; Neu, neutrophil; Plt, platelet; PT‐INR, prothrombin time‐international normalized ratio; RBC, red blood cell; sIL‐2R, soluble IL‐2 receptor.T‐Bil, total bilirubin; TP, total protein; UA, uric acid; WBC, white blood cell; γ‐GTP, γ‐glutamyl transpeptidase.

## 3 DISCUSSION

Venetoclax is an orally bioavailable, selective, small‐molecule inhibitor of BCL2 that is highly effective for relapsed or refractory CLL; however, TLS is reported to occur as a severe adverse event of venetoclax. The risk of TLS increases with renal dysfunction, and venetoclax use has not been studied in patients with severe renal impairment. In the phase three study, CLL patients with moderate renal impairment (creatinine clearance <50 ml/min) were excluded,^2^ and the safety of venetoclax for patients with severe CKD (stage 4, GFR < 30 ml/min) was not elucidated. To the best of our knowledge, this is the first case in which venetoclax was used for a CLL patient with severe CKD who was not dependent on dialysis. A case report revealed that venetoclax was safely administered in a multiple myeloma patient with renal failure who was dependent on dialysis,^5^ but this patient had undergone dialysis before venetoclax administration. The clinical situation of that case was completely different from that in this case.

Although we considered the dose and schedule about administration of venetoclax to the patient with severe CKD, we decided to administer venetoclax with no dose reduction and no extension of the treatment schedule because venetoclax and its major metabolite are primarily metabolized by the liver cytochrome P450 3A4 enzyme, and urinary clearance is negligible.^6^ In addition, a 50% dose reduction for patients with severe liver impairment is recommended,^7^ but this patient had only mild liver impairment due to CLL, we assessed that no dose reduction for liver function is needed.

Fortunately, venetoclax was safely administered without the onset of TLS in this case. Debulking by rituximab and cyclophosphamide with prednisolone was successful, and it seemed to reduce TLS risk. In addition, intensive prevention of TLS using hydration, febuxostat, and rasburicase as uric acid‐reducing agents seemed to be important too. However, TLS is sometimes inevitable and dialysis should be initiated without hesitation if TLS is suspected. Thus, strict monitoring of laboratory data also seemed to be important.

In conclusion, we describe the first case of a relapsed CLL patient with severe CKD due to congenital solitary kidney that was successfully treated with venetoclax. Venetoclax was safely administered by intensive prevention and strict monitoring of TLS. This report may suggest safety and efficacy about using venetoclax in CLL patients with severe CKD, only if proper risk management of TLS is given.

## CONFLICT OF INTEREST

The authors declare no potential conflicts of interest regarding the publication of this study.

## AUTHOR CONTRIBUTIONS

H.S. managed the clinical practice and authored this case study. N.S. managed and supervised the clinical practice. T.I., T.K., S.O., N.N., T.M., and Y.N. provided advice on the paper. T.K. and M.T. supervised the clinical practice.

## ETHICAL APPROVAL

Because all medical care and examination were provided by public health insurance in Japan, this case report was not deemed to be the subject which needed IRB oversight in our institution.

## CONSENT

Written informed consent has been obtained from the patient.

## PERMISSION TO REPRODUCE MATERIAL FROM OTHER SOURCES

The authors do not borrow any materials from other works.

## CLINICAL TRIAL REGISTRATION

The patient did not registrate any clinical trial.

## Data Availability

The data during this study are available from the corresponding author upon reasonable request.
